# MALIGNANT ECCRINE SPIRADENOMA ON THE LATERAL MARGIN OF NOSE AS AN INFREQUENT LOCALIZATION

**DOI:** 10.4103/0019-5154.53191

**Published:** 2009

**Authors:** Sami Berçin, Ahmet Kutluhan, Ahmet Metin, Dinç Süren

**Affiliations:** *From the Department of Otolaryngology, Atatürk Training and Research Hospital, Ankara, Turkey*; 1 *From the Department of Dermatology, Atatürk Training and Research Hospital, Ankara, Turkey*; 2 *From the Department of Pathology, Atatürk Training and Research Hospital, Ankara, Turkey*

**Keywords:** *Skin tumour*, *eccrine spiradenoma*, *malignant*

## Abstract

Malignant eccrine spiradenomas are exceedingly rare tumors. They can arise from a preexisting eccrine spiradenoma or occur as a primary malignant tumor. Clinical features of these tumors may include a history of enlargement in a previously stable lesion. Tumor can be of low or high grade. Low-grade malignant eccrine spiradenoma has a better prognosis. We present a malignant eccrine spiradenoma arising from a preexisting eccrine spiradenoma, which has an infrequent localization between lateral edge of nose and medial canthus.

## Introduction

Malignant eccrine spiradenomas are exceedingly rare tumors. They can arise from a preexisting eccrine spiradenoma or occur as a primary malignant tumor.[1] Clinical features of these tumors may include a history of enlargement in a previously stable lesion. Tumor can be of low or high grade. Low-grade malignant eccrine spiradenoma has a better prognosis. We present a malignant eccrine spiradenoma arising from a preexisting eccrine spiradenoma, which has an infrequent localization between lateral edge of nose and medial canthus.

## Case Report

**Figure 1:**
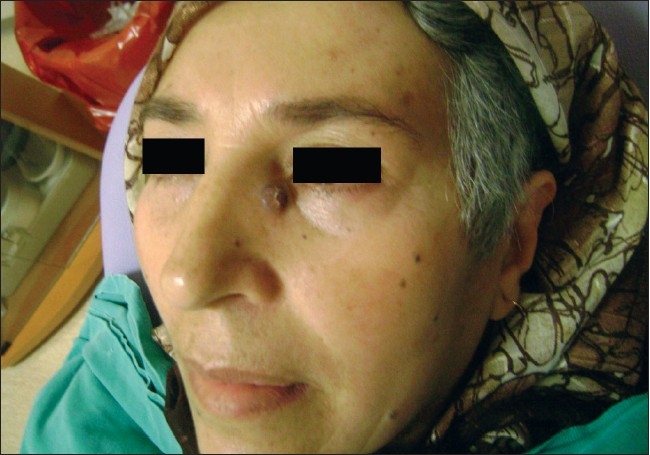
Appearence of the lesion on lateral part of nose and anteriorinferior aspect of medial canthus

**Figure 2:**
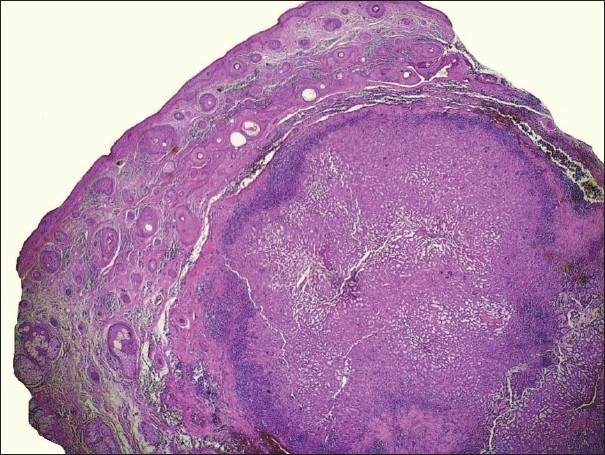
Nodular tumor located in dermis. Note that the heavy infiltration by lymphocytes is mainly present at the peripheral portions of the tumor (H&E, ×20)

A 64-year-old female patient was admitted to our department, with a 1 × 1 cm swelling on her upper lateral aspect of her nose, which had been present for 6 months. She had noticed a sudden enlargment of the painless, dark pink-blue noduler lesion on her nose for the past two months [Figure 1]. There was no palpable lymph node in the neck. The lesion was excised with a 5 mm free margin and the wound was closed by primary suturation. Gross examination of the resected specimen showed a 1 × 1 cm, pink–blue noduler lesion. The specimen was fixed in a 10% neutral buffered formaldehyde solution and embedded in paraffin. It was routinely processed with hematoxylin and eosin staining. Microscopic examination revealed a nodular tumor located in the dermis. The overlying epidermis demonstrated hyperplastic changes. A heavy lymphocytic infiltration was present mainly at the peripheral portions of the tumor, but some were also scattered between the tumor cells [Figure 2].

**Figure 3:**
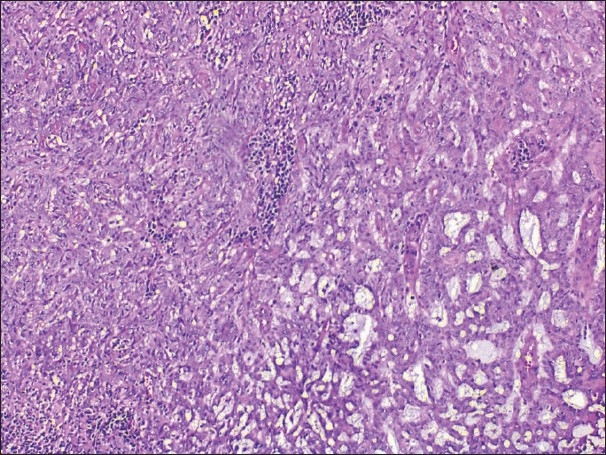
Solid areas of the tumor composed of malignant cells with nuclear atypia and high mitotic figures (left). The other component resembled an ecrine spiradenoma; with two distinct cell proliferation and tubule formations (right) (H&E, ×100)

At higher magnifications, two distinct populations of neoplastic epithelial cells (small dark basaloid cells and pale cells, which are larger with vesicular nuclei and pale cytoplasm) and tubule formations were characteristic features of the tumor. Tubules lined by two rows of epithelial cells were found within the tumor. The presence of basaloid secretion material, some surrounded by neoplastic cells, throughout the neoplasm attracted attention [Figure 3].

**Figure 4:**
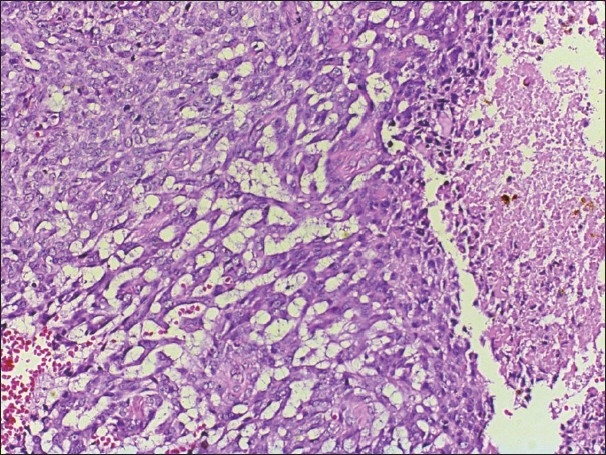
Focal tumor cell necrosis is seen through out the tumor (H&E, ×200)

The resulting appereance resembles an eccrine spiradenoma. However in some areas, the tumor showed malignant change. These malignant components were composed of solid aggregates of tumor cells with mild to modarate nuclear atypia, high mitotic figures (12/10 high-power field) and focal tumor cell necrosis [Figures 3 and 4]. Vascular invasion was not present. The surgical margins of excision were free of tumor. The pathologic findings were concordant with transformation to malignant eccrine spiradenoma because of the absence of palpable or ultrasonographic lymph node in head and neck and metastasis in adjacent structures like paranasal sinuses. Close follow-up was initiated.

## Discussion

Eccrine spiradenomas are benign adnexal tumors with slow growth and long clinical history. Malignant eccrine spiradenomas (MES) are rare but have been divided into two categories: Those arise *de novo* and those develop from a benign eccrine spiradenoma.[2] The first MES was described in 1972 by Dabska. Since then, nearly 50 cases have been described in the literature.[1] Typically, sudden growth is noted in a tumor that has been present for many years or even decades.[2] Clinical features of MES reported in the literature include a previously stable lesion of more than 2 years of duration in 87.5% of patients and of more than 12 years duration in 66.7%.[3] There is no age, gender, or site predilection.[4] Large majority of cases have occurred on the extremities or trunk and very rare cases have been reported on the head and neck.[1] In our case, the lesion had an infrequent localization; on the upper part of nose, anterior inferior of left medial canthus. The lesion was present for 6 months and had shown a rapid growth for 2 months. Rapid growth of the tumor was the presenting symptom in our patient.

Granter *et al.*, in their study of 12 MES patients, reported equal sex incidence and an average age of occurrence at 62 years.[5] Seven tumors were located on the trunk, three on the extremities, and two in the head and neck region. Average size of the tumors was 7.5 cm. Lesions had been present from 7 months to 30 years before surgical removal. Five of seven patients on whom there was follow-up information were free of disease, and one patient developed metastases to local lymph nodes 5 years after the primary tumor was resected. Average duration of follow-up was 3.4 years.[5] Tay *et al.*, reported 57% local recurrance and 39% metastasis and related death in MES.[6] Leonard *et al.*, reported 8 of 9 cases of MES in literature are high grade criteria with nuclear pleomorphism, high mitotic count and abnormal mitoses.[2] More than half of the case reports mention abnormal cell diferentiation with squamous and sarcomatous areas. The patients died with lung, brain, liver, bone, and skin metastasis after 5 months to 11 years.[2] Survival for 30 months after diagnosis of low-grade MES with metastasis was reported by the same author. Leonard *et al.*,[1] stated that biologic behavior of the tumor is nonspecific. Because of high metastatic capacity of the tumor, it is considered to be potentially lethal. In the literature, MES is reported to be an aggresive tumor with a mortality rate between 20 and 39%.[4,7] MES is a histologically heterogenous tumor and, it has two distinct paterns; high-grade carcinoma and low-grade tumor imitating benign spiradenoma. Pathologists must examine all eccrine spiradenomas carefully in view of malignant transformation. Histologic hallmarks of MES are proliferation of solid masses of tumor cells with large hyperchromatic nuclei and frequent mitoses. Other features of the tumor are invasion of surrounding connective tissue, loss of basement membrane, and squamous differentiation.[8] Mitotic rates are between 4 and 32 per HPF (high power field) in high grade lesions, and between 2 and 12 in low-grade lesions.[2] In histopathological examination of our case, features suggesting eccrine spiradenoma and also MES are found. Medium grade nuclear atypia, relatively lower mitotic count, not much pleomorphism, lack of carcinomatous and sarcomatous areas, and vascular invasion suggest that this lesion is low-grade MES.

The clinical course of MES varies in the reported cases. Metastasis of bones, lungs, brain, and lymph nodes have been described.[9,10] Close postoperative follow-up is very important to monitor local recurrence, or regional or distant metastasis. Wide local excision seems to be the adequate treatment for MES. Role of prophylactic lymph node dissection, postoperative adjuvant radiotherapy, or chemotheraphy is uncertain because of limited data and follow-up time.[8] If there is an absence of distant metastasis and presence of regional lymph node metastasis, lymph node dissection should be undertaken.[8] Our patient has tumor free margin, no palpable lymph node in the neck or evidence of lympadenopathy on ultrasonographic evaluation of neck and no distant metastasis; therefore, she was not put on any adjuvant therapy.

Rapid changes of appearence of the benign skin lesions, rapid growth, color change, and ulcerations are clues to the malignant transformation. Therefore, they must be followed up carefully. In conclusion, we need more information about MES, its biological behavior, and treatment modalities.

## References

[1] Beekley AC, Brown TA, Porter C (1999). Malignant eccrine spiradenoma: A previously unreported presentation and review of the literature. Am Surg.

[2] Leonard N, Smith D, McNamara P (2003). Low grade Malignant eccrine spiradenoma with systemic metastases. Am J Dermatol.

[3] Elder D, Elenitsas R, Jaworsky C (1997). Lever's Histopathology of the skin.

[4] Ishikawa M, Nakanishi Y, Yamazaki N (2001). Malignant eccrine spiradenoma: A case report and review of the literature. Dermatol Surg.

[5] Granter SR, Seeger K, Calonje E (2000). Malignant Eccrine spiradenoma (Spiradenocarcinoma): A clinicopathologic study of 12 cases. Am J Dermatopathol.

[6] Tay JS, Tapen EM, Solari PG (1997). Malignant eccrine spiradenoma, case report and review of the literature. Am J Clin Oncol.

[7] Yildirim S, Aköz T, Akan M De Novo. Malignant Eccrine Spiradenoma with and Interesting and Unusual Location.

[8] Jamshidi M, Novak MA, Chiu YT (1999). Giant Eccrine spiradenoma of the scalp. Dermatol Surg.

[9] Evans H, Su WP, Smith JL (1979). Carcinoma arising in eccrine spiradenoma. Cancer.

[10] Zamboni AC, Zamboni WA, Ross DS (1990). Malignant eccrine spiradenoma of the hand. J Surg Oncol.

